# Transition in metabolic health phenotypes across general adiposity categories and association with the risk of depression: a prospective analysis

**DOI:** 10.1192/j.eurpsy.2024.20

**Published:** 2024-02-29

**Authors:** Yunyi Zhu, Yuan Yin, Fei Huang, Yuanjun Liu, Yuge Xia, Mingying Zhang, Yunhe Wang, Lijuan Jin

**Affiliations:** 1 Suzhou Hospital of Traditional Chinese Medicine, Endocrinology and Metabolic Diseases Department, Suzhou, China; 2 The Second Affiliated Hospital of Anhui University of Chinese Medicine, Geriatric Department, Hefei, China; 3 Nuffield Department of Population Health, University of Oxford, Oxford, UK

**Keywords:** body mass index, depression, metabolic health

## Abstract

**Background:**

The association between obesity and depression may partly depend on the contextual metabolic health. The effect of change in metabolic health status over time on subsequent depression risk remains unclear. We aimed to assess the prospective association between metabolic health and its change over time and the risk of depression across body mass index (BMI) categories.

**Methods:**

Based on a nationally representative cohort, we included participants enrolled at the wave 2 (2004–2005) of the English Longitudinal Study of Ageing and with follow-up for depression at wave 8 (2016–2017). Participants were cross-classified by BMI categories and metabolic health (defined by the absence of hypertension, diabetes, and hypercholesterolemia) at baseline or its change over time (during waves 3–6). Logistic regression model was used to calculate odds ratios (ORs) and 95% confidence intervals (CIs) for the risk of depression at follow-up stratified by BMI category and metabolic health status with adjustment for potential confounders.

**Results:**

The risk of depression was increased for participants with metabolically healthy obesity compared with healthy nonobese participants, and the risk was highest for those with metabolically unhealthy obesity (OR 1.62, 95% CI 1.18–2.20). Particularly hypertension and diabetes contribute most to the increased risk. The majority of metabolically healthy participants converted to unhealthy metabolic phenotype (50.1% of those with obesity over 8 years), which was associated with an increased risk of depression. Participants who maintained metabolically healthy obesity were still at higher risk (1.99, 1.33–2.72), with the highest risk observed for those with stable unhealthy metabolic phenotypes.

**Conclusions:**

Obesity remains a risk factor for depression, independent of whether other metabolic risk factors are present or whether participants convert to unhealthy metabolic phenotypes over time. Long-term maintenance of metabolic health and healthy body weight may be beneficial for the population mental well-being.

## Introduction

Obesity and depression are major causes of death and disability [1, 2]. Previous evidence based on meta-analysis of observational studies found that obesity and overweight may contribute to an increased risk of depression [3], although inconsistent findings were also reported [4, 5]. It has been suggested that the adverse health impact of obesity may be partly dependent on the contextual metabolic health [6]. For example, a proportion of obese individuals may not exhibit cardiometabolic abnormalities (e.g., hypertension, hyperglycemia, and hypercholesterolemia), a phenotype referred to as metabolically healthy obesity [6, 7]. However, the adverse health impact of metabolically healthy obesity on mental well-being remains controversial. According to the limited evidence from several cross-sectional studies, obese participants with unhealthy metabolic profiles were generally at increased risk of depression compared with nonobese healthy participants, whereas the association with metabolically healthy obese was inconsistent, with a similar or increased depression risk reported [4, 5, 8]. Metabolic status is also relevant for nonobese individuals. It is estimated that 15% of individuals, despite having a normal weight, had metabolic disorders [7]. Metabolically unhealthy nonobese was reported to be associated with comparable or slightly lower risk of depression compared with metabolically unhealthy obese [4, 5].

These differences in findings across studies could be partly attributed to the varying definitions of metabolic health components and criteria employed [9], thus complicating the comparison and interpretation of the results. In addition, previously used definitions mainly allow for one or more metabolic risk factors to be present at one time point, which may not be enough to identify obese individuals truly with low metabolic risk [6]. By contrast, using a more strict definition, as the absence of major metabolic diseases (e.g., hypertension, diabetes, and hypercholesterolemia), may better identify a metabolically healthy obese phenotype and estimate the association of interest [7, 10]. Further, considering the dynamic nature of metabolic phenotype across adiposity categories, possibly influenced by lifestyle behavior, aging, and health-related conditions, studies with long-term follow-up are needed to assess the health impact of transition in obesity phenotypes. The risk of adverse health outcomes for metabolically healthy obese was higher in studies with longer follow-up, suggesting metabolically healthy obesity represents a transient phenotype and may transfer to metabolically unhealthy status over time [10, 11]. Notably, 33–84% of metabolically healthy obese individuals converted to an unhealthy metabolic phenotype over 6–20 years, and 64% of individuals with healthy normal weight transitioned to metabolically unhealthy nonobese phenotype over 20 years in previous prospective cohort studies [7]. Nevertheless, the potential changing patterns of metabolic health status across adiposity categories over time and their relevance to subsequent risk of depression remain unclear.

Based on a nationally representative community-based cohort, we aimed to assess the association between metabolic health (defined as the absence of diagnosed hypertension, diabetes, and hypercholesterolemia) and its change over time and subsequent risk of depression across general adiposity categories as measured by BMI. We also investigated the association between the components of metabolic risk factors and depression risk across BMI categories. We hypothesize that metabolically unhealthy obesity at baseline is related to a higher risk of depression and the transition from healthy to unhealthy metabolic phenotypes over time also increases depression risk.

## Methods

### Study population

The English Longitudinal Study of Ageing (ELSA) is an ongoing nationally representative cohort study that recruited adults aged 50 years and older living in private households in England, as detailed elsewhere [7]. Briefly, the original sample included 11,391 participants from the Health Survey for England (HSE) in 1998, 1999, and 2001 (wave 0), and the first wave of data collection took place on March 1, 2002 (wave 1), with subsequent longitudinal assessments every 2 years to measure changes in the health, economic and social circumstances using face-to-face interviews and self-administered questionnaires, and additional nurse visits every 4 years from the wave 2. Further refreshment samples based on HSE at several waves (waves 3, 4, 6, 7, and 9) with different age criteria were included to correct for the age profile as the original sample aged. For the aims of the current analyses, we used wave 2 (2004–2005) as the study baseline, because of the more extensive assessment of metabolic health, BMI, and covariates. Follow-up for depression was made 12 years after based on the data collected at wave 8 (2016–2017). All participants provided written consent and ethical approval for all waves of the ELSA study was granted by NHS Research Ethics Committees under the National Research and Ethics Service. This longitudinal study is based on data from the waves 2–6 and 8 of the ELSA. We excluded participants who were underweight and had missing data on BMI at wave 2, and those who did not attend the wave 8 follow-up. Detailed study design and timelines are provided inFigure S1 in the Supplementary Material.

### Measurements

Height, weight, and waist circumference were measured by a trained nurse at wave 2. Height was measured with shoes using a portable stadiometer positioned horizontally according to the Frankfort plane. Weight was measured using a portable electronic scale. Waist circumference was measured twice, taking the midpoint between the lower rib and the upper margin of the iliac crest. If the difference between the first two waist circumference measurements was within 3 cm, their mean values were used; otherwise, a third measurement was obtained, and the two closest results were used to calculate the mean. Body mass index (BMI) was derived by dividing weight in kilograms by the square of height in meters. Two sets of BMI categories were used: we first classified participants into normal weight (BMI 18.5–24.9 kg/m^2^), overweight (BMI 25.0–29.9 kg/m^2^), and obesity (BMI ≥30 kg/m^2^); and then classified participants into nonobese (BMI 18.5–29.9 kg/m^2^) and obesity (BMI ≥30 kg/m^2^). Metabolic health was defined based on existing criteria [12] using metabolic disorders hypertension, diabetes, and hypercholesterolemia. Participants with at least one of these disorders were considered to be metabolically unhealthy and those with none of these disorders were considered to be metabolically healthy. Information on metabolic health was based on self-reported physician-diagnosed diseases on questionnaires updated biennially from wave 2.

Depressive symptoms were assessed at baseline and follow-up by an abbreviated 8-item version of the validated Center for Epidemiologic Studies Depression Scale (CES-D 8). As in previous studies, a score of ≥4 was used to define participants with elevated depressive symptoms [13]. The CES-D is validated to have excellent psychometric properties for use as a screening tool for major depression in older adults [14, 15]. It should be noted that the CES-D is not a diagnostic instrument for clinical depression.

Covariates including age, sex, race/ethnicity, education level, smoking, alcohol consumption, physical activity, diagnosed cardiovascular diseases, and history of psychiatric disorders were measured by questionnaires at baseline and updated every 2 years.

### Statistical analyses

Logistic regression model was used to calculate odds ratios (ORs) and 95% confidence intervals (CIs) for the risk of depression at follow-up stratified by BMI category and metabolic health status. We adjusted for the covariates at baseline including age, sex (men and women), race/ethnicity (white and others), education level (lower than high school, high-school graduate, some college, and college and above), smoking (never, ever, and current smoker), alcohol consumption (frequency of drinking), physical activity (frequency of vigorous physical activity), cardiovascular diseases (yes or no), history of psychiatric disorders (yes or no), and CES-D score. The interaction between BMI and metabolic health on depression risk was tested. To assess the effect of long-term changes in metabolic health status, we further evaluated the risk of depression stratified for metabolic health at baseline (wave 2, 2004–2005) and at 8-year follow-up (wave 6, 2012–2013), with follow-up for depression started at wave 6 and adjusted for wave 6 CES-D score. To assess the effect of individual component of metabolic health on risk of depression, we evaluated the depression stratified for hypertension, diabetes, and hypercholesterolemia and BMI, with adjustment for covariates and mutually for other metabolic diseases. To examine the potential statistical interaction, prespecified subgroup analyses by age (<65 vs. ≥65 years), sex (male and female), and smoking status (ever smokers and never smokers) were conducted for depression risk across metabolic health status and BMI categories.

Several sensitivity analyses mainly to rule out reverse causation caused by unhealthy normal weight were conducted to assess the robustness of the main analyses: exclusion of participants with history of diagnosed mental disorders at baseline; further adjustment for other chronic diseases at baseline (e.g., cardiovascular diseases, lung disease, asthma, arthritis, osteoporosis, and cancer); using data collected at wave 9 (2018–2019) for follow-up of depression; further adjustment for central obesity (as defined by waist circumference); further adjustment for weight change between baseline at wave 2 and wave 6 follow-up. Finally, as the lack of association of metabolically healthy obesity with depression in previous studies may be attributable to the shorter time period of exposure to obesity, we excluded participants who were metabolically healthy obese at baseline but were not already obese about 4–5 years ago at wave 0.

All analyses were performed using SAS version 9.4 (SAS Institute) and R version 4.2.2 (R Foundation), and all statistical tests were 2-sided, with *p* < 0.05 considered significant.

## Results

### Population characteristics

Approximately 12,397 participants attended the wave 2 (baseline, 2004–2005) survey, of which 7,225 participants took part in the nurse interview with BMI measured. After excluding those who were underweight and with missing data on BMI, 3,397 participants with follow-up for depression at wave 8 (2016–2017) were included in the current study (Figure S1 in the Supplementary Material). Baseline characteristics of eligible participants stratified by BMI categories and metabolic health status are shown in Table 1. Approximately 29.7% of participants were obese at baseline, whereas 43.9% of this group was metabolically healthy at baseline. Across BMI categories (nonobese and obese), metabolically unhealthy participants had higher age and prevalence of comorbidities (e.g., lung, cardiovascular diseases, asthma, and arthritis), and lower frequency of vigorous physical activity than those who were metabolically healthy. The mean BMI and WC were only slightly higher among metabolically unhealthy participants within BMI categories of nonobese (BMI difference by 0.6 kg/m^2^ and WC difference by 2.4 cm) and obese (by 0.8 kg/m^2^ and 1.9 cm).

**Table 1.** Population characteristics by BMI categories and metabolic health status at baseline

	Overall	Nonobese		Obese	
		Metabolically healthy	Metabolically unhealthy	Metabolically healthy	Metabolically unhealthy
No.	3,397	1,448 (42.6%)	942 (27.7%)	441 (13.0%)	566 (16.7%)
BMI (kg/m^2^)	28.1 (4.9)	25.4 (2.6)	26.0 (2.5)	33.6 (3.6)	34.4 (4.2)
Age (years)	62.7 (7.2)	61.9 (7.0)	64.2 (7.5)	61.3 (7.1)	63.2 (7.1)
Male	1,463 (43.1)	648 (44.8%)	437 (46.4%)	166 (37.6%)	212 (37.5%)
White ethnicity	3,338 (98.3%)	1,431 (98.8%)	922 (97.9%)	433 (98.2%)	552 (97.5%)
Education level of college and above	543 (16%)	286 (19.8%)	138 (14.6%)	52 (11.8%)	67 (11.8%)
Current or former smoker	2,013 (59.3%)	841 (58.1%)	553 (58.7%)	266 (60.3%)	353 (62.4%)
Alcohol consumption (at least one drink per week)	2,538 (74.7%)	1,151 (79.5%)	714 (75.8%)	303 (68.7%)	370 (65.4%)
Vigorous physical activity (at least once per week)	820 (24.1%)	411 (28.4%)	234 (24.8%)	91 (20.6%)	84 (14.8%)
Metabolic health status					
Hypertension	1,176 (34.6%)	0	701 (74.4%)	0	475 (83.9%)
Diabetes	186 (5.5%)	0	88 (9.3%)	0	98 (17.3%)
Hypercholesterolemia	613 (18.0%)	0	400 (42.5%)	0	213 (37.6%)
Systolic blood pressure (mm Hg)	133.2 (17.7)	128.0 (16.6)	137.1 (17.3)	134.8 (17.7)	138.7 (17.6)
Diastolic blood pressure (mm Hg)	76.1 (10.4)	74.0 (9.7)	76.4 (10.6)	78.3 (9.7)	78.8 (11.0)
Fasting glucose level (mmol/L)^a^	4.98 (0.8)	4.87 (0.7)	5.04 (0.8)	5.03 (0.8)	5.18 (1.0)
Comorbidity					
Chronic lung diseases	138 (4.1%)	53 (3.7%)	42 (4.5%)	15 (3.4%)	28 (4.9%)
Cardiovascular diseases	381 (11.2%)	136 (9.4%)	121 (12.8%)	35 (7.9%)	89 (15.7%)
Asthma	415 (12.2%)	157 (10.8%)	112 (11.9%)	56 (12.7%)	90 (15.9%)
Arthritis	1,059 (31.2%)	346 (23.9%)	295 (31.3%)	160 (36.3%)	258 (45.6%)
Osteoporosis	124 (3.7%)	61 (4.2%)	39 (4.1%)	6 (1.4%)	18 (3.2%)
Psychiatric disorders	131 (3.9%)	48 (3.3%)	33 (3.5%)	22 (5.0%)	28 (4.9%)
Waist circumference (cm)	95.3 (12.9)	89.2 (10.1)	91.6 (9.8)	106.5 (10.1)	108.4 (10.5)
BMI categories at wave 0					
Nonobese	2,603 (76.4%)	1,407 (97.2%)	899 (95.4%)	141 (32.0%)	156 (27.6%)
Obese	794 (23.6%)	41 (2.8%)	43 (4.6%)	300 (68.0%)	410 (72.4%)
BMI change between baseline and wave 6 (kg/m^2^)	0.36 (2.28)	0.47 (1.89)	0.48 (1.93)	0.17 (2.67)	0.02 (3.20)

Data are mean (SD) or *n* (%).

a Data are available in a subsample of participants with blood sample collected.

### Metabolic health, BMI, and risk of depression

The risk of depression cross-classified by BMI categories and metabolic health status are shown in Table 2. The risk of depression was higher in metabolically unhealthy participants across BMI categories. Compared with metabolically healthy nonobese participants, both the metabolically healthy obese (OR, 1.42; 95% CI, 1.00–2.02) and unhealthy obese (1.62, 1.18–2.20) participants were at an increased risk of depression at 12 years of follow-up after adjustment for baseline CES-D score and other covariates. We further divided the nonobese into normal weight and overweight. The results similarly suggested that, compared to participants with metabolically healthy normal weight, both metabolically healthy and unhealthy obese were associated with an increased risk of depression; and participants with metabolically unhealthy overweight had an increased depression risk (1.51, 1.02–2.25) as did those with metabolically healthy obese (1.57, 1.03–2.41). We found no significant interaction between BMI and metabolic health status on the risk of depression (*p* = 0.67). We then tested the association between individual components of metabolic health and depression. The trend of increased risk of depression was observed for obese participants with or without hypertension and diabetes, whereas hypercholesterolemia had a relatively lower effect on depression risk for participants who were obese at baseline (Table 3).

**Table 2.** Association between general obesity, metabolic health, and risk of depression

		OR (95% CI)	
BMI^a^ and metabolic health^b^	Events/*N* (%)	Age- and sex-adjusted model	Multivariable-adjusted mode^c^
Category 1			
Metabolically healthy nonobese	129/1,448 (8.9%)	1 [Reference]	1 [Reference]
Metabolically unhealthy nonobese	121/942 (12.8%)	1.39 (1.06–1.82)	1.26 (0.95–1.68)
Metabolically healthy obese	59/441 (13.4%)	1.55 (1.11–2.15)	1.42 (1.00–2.02)
Metabolically unhealthy obese	99/566 (17.5%)	1.98 (1.48–2.63)	1.62 (1.18–2.20)
Category 2			
Metabolically healthy normal weight	53/601 (8.8%)	1 [Reference]	1 [Reference]
Metabolically unhealthy normal weight	33/297 (11.1%)	1.18 (0.73–1.86)	1.16 (0.70–1.90)
Metabolically healthy overweight	76/847 (9.0%)	1.12 (0.78–1.63)	1.19 (0.80–1.78)
Metabolically unhealthy overweight	88/645 (13.6%)	1.64 (1.14–2.38)	1.51 (1.02–2.25)
Metabolically healthy obese	59/441 (13.4%)	1.65 (1.11–2.47)	1.57 (1.03–2.41)
Metabolically unhealthy obese	99/566 (17.5%)	2.11 (1.48–3.04)	1.79 (1.22–2.65)

a BMI category 1: normal weight (BMI 18.5–24.9 kg/m^2^), overweight (BMI 25.0–29.9 kg/m^2^), and obesity (BMI ≥30 kg/m^2^); BMI category 2: nonobese (BMI 18.5–29.9 kg/m^2^) and obesity (BMI ≥30 kg/m^2^).

b Metabolic health status was based on the presence of metabolic disorders hypertension, diabetes, and hypercholesterolemia.

c Multivariate model was adjusted for age, sex, race/ethnicity, education level, smoking, alcohol consumption, physical activity, cardiovascular diseases, history of psychiatric disorders, and CES-D score at baseline.

**Table 3.** Association between individual component of metabolic risk factors at baseline and risk of depression

	Nonobese		Obese	
	No individual metabolic risk factor	Individual metabolic risk factor	No individual metabolic risk factor	Individual metabolic risk factor
Hypertension				
Events/*N* (%)	156/1,689 (9.2%)	94/701 (13.4%)	72/532 (13.5%)	86/475 (18.1%)
OR, age– and sex–adjusted^a^	1 [Reference]	1.36 (1.02–1.80)	1.50 (1.10–2.02)	1.83 (1.35–2.46)
OR, multivariate–adjusted^b^	1 [Reference]	1.23 (0.91–1.65)	1.35 (0.97–1.86)	1.56 (1.12–2.16)
Diabetes				
Events/*N* (%)	236/2,302 (10.3%)	14/88 (15.9%)	136/909 (15.0%)	22/98 (22.4%)
OR, age– and sex–adjusted^a^	1 [Reference]	1.68 (0.88–2.99)	1.44 (1.14–1.81)	2.15 (1.26–3.53)
OR, multivariate–adjusted^b^	1 [Reference]	1.36 (0.68–2.56)	1.32 (1.02–1.69)	1.75 (0.98–3.00)
Hypercholesterolemia				
Events/*N* (%)	204/1,990 (10.3%)	46/400 (11.5%)	116/794 (14.6%)	42/213 (19.7%)
OR, age– and sex–adjusted^a^	1 [Reference]	1.03 (0.72–1.44)	1.38 (1.08–1.77)	1.77 (1.20–2.58)
OR, multivariate–adjusted^b^	1 [Reference]	1.04 (0.71–1.48)	1.34 (1.02–1.75)	1.28 (0.83–1.94)

a Adjusted for age, sex, and mutually for other metabolic risk factors at baseline.

b Adjusted for age, sex, race/ethnicity, education level, smoking, alcohol consumption, physical activity, cardiovascular diseases, history of psychiatric disorders, CES-D score, and mutually for other metabolic risk factors at baseline.

Predefined subgroup analyses suggested no significant interaction for age, sex, and smoking (Table S1 in the Supplementary Material). Sensitivity analysis excluding participants with diagnosed mental disorders at baseline indicated consistent results for the association between BMI, metabolic health, and depression (Table S2 in the Supplementary Material). Further adjustment for other chronic diseases, central obesity, and weight change between baseline and wave 6, and using wave 9 follow-up data for depression did not materially alter the results. As previous studies suggested that metabolically healthy obesity may be a transient status and their health impact may be related to the time period of exposure to obesity [4, 7], we further excluded those who were metabolically healthy obese at baseline but were not already obese about at wave 0. We found that 73.5% of the metabolically healthy obese participants at baseline were in fact classified as obese already 4–5 years ago. The risk of depression for participants being stable metabolically healthy obesity was stronger after exclusion compared with those with metabolically healthy nonobese (1.57, 1.06–2.30).

### Change in metabolic health, BMI, and risk of depression

We further assessed the effect of changes in metabolic health status over time on depression across BMI categories. For participants with metabolic health at baseline, most converted to metabolically unhealthy over a median of 8 years in all BMI categories (205 [34.1%] of 601 participants with normal weight, 328 [38.7%] of 847 with overweight, and 221 [50.1%] of 441 with obesity). Table 4 presents the risk of depression cross-classified by BMI categories and metabolic health status at baseline and follow-up. Within each BMI category, the risk of depression was higher for those who were metabolically unhealthy at baseline and became so over time. Participants with metabolically healthy obesity at baseline who stayed metabolically healthy throughout 8 years were still at increased risk of depression compared to those with stable metabolically healthy nonobese (OR, 1.66; 95% CI, 1.00–2.73), which was slightly lower than initially metabolically healthy participants who converted to unhealthy metabolic phenotype (1.68, 1.02–2.71). The risk of depression was highest for those with stable unhealthy metabolic phenotypes across BMI categories. Sensitivity analyses excluding those with a history of diagnosed mental disorders, additional adjustment for other chronic diseases at baseline, central obesity, and weight change between baseline and follow-up, and using wave 9 follow-up data for depression indicated consistent results (Table S2 in the Supplementary Material).

**Table 4.** Association between general obesity, metabolic health at baseline (2004/2005) and at follow-up (2012/2013), and risk of depression

a, Category 1						
	Nonobese at baseline			Obese at baseline		
	Metabolically healthy at baseline			Metabolically healthy at baseline		
	Metabolically healthy at follow–up	Metabolically unhealthy at follow–up	Metabolically unhealthy at baseline	Metabolically healthy at follow–up	Metabolically unhealthy at follow–up	Metabolically unhealthy at baseline
Events/*N* (%)	67/915	62/533	121/942	27/220	32/221	99/566
	(7.3%)	(11.6%)	(12.8%)	(12.3%)	(14.5%)	(17.5%)
OR (95% CI)^a^	1 [Reference]	1.47 (1.00–2.17)	1.49 (1.06–2.09)	1.66 (1.00–2.73)	1.68 (1.02–2.71)	1.90 (1.33–2.72)

b, Category 2									
	Normal weight at baseline			Overweight at baseline			Obese at baseline		
	Metabolically healthy at baseline			Metabolically healthy at baseline			Metabolically healthy at baseline		
	Metabolically healthy at follow–up	Metabolically unhealthy at follow–up	Metabolically unhealthy at baseline	Metabolically healthy at follow–up	Metabolically unhealthy at follow–up	Metabolically unhealthy at baseline	Metabolically healthy at follow–up	Metabolically unhealthy at follow–up	Metabolically unhealthy at baseline
Events/*N* (%)	32/396	21/205	33/297	35/519	41/328	88/645	27/220	32/221	99/566
	(8.1%)	(10.2%)	(11.1%)	(6.7%)	(12.5%)	(13.6%)	(12.3%)	(14.5%)	(17.5%)
OR (95% CI)^a^	1 [Reference]	1.13 (0.60–2.10)	1.22 (0.70–2.12)	0.97 (0.57–1.66)	1.69 (1.00–2.89)	1.59 (1.01–2.89)	1.64 (0.91–2.94)	1.66 (0.94–2.92)	1.88 (1.20–3.00)

a ORs adjusted for age, sex, race/ethnicity, education level, smoking, alcohol consumption, physical activity, cardiovascular diseases, history of psychiatric disorders, and CES-D score at baseline.

## Discussion

Based on a nationally representative cohort, we found that both obesity and unhealthy metabolic conditions are independently associated with an increased risk of depression. Participants with both metabolically healthy obesity and metabolically unhealthy obesity were at an increased risk compared with those with metabolically healthy nonobese or normal weight. Our results further suggested that a large proportion of metabolically healthy participants converted to unhealthy metabolic phenotypes over time across BMI categories of normal weight, overweight, and obesity, which was associated with an increased risk of depression. The highest risk of depression was observed for participants with persistent metabolically unhealthy obesity. However, even when metabolic health was maintained over 8 years, metabolically healthy obesity was still associated with an increased risk of depression compared with stable nonobese. Overall, our study supported that obesity remains a risk factor for depression, independent of whether other metabolic risk factors are present or whether participants convert to unhealthy metabolic phenotypes over time. These findings suggested the importance of long-term maintenance of metabolic health and healthy body weight for the population mental well-being. Considering the stable healthy metabolic phenotype is difficult to maintain across all BMI categories over long periods of time, targeted behavior (such as adherence to a healthy diet and physical activity) and medical interventions preventing the transition to being metabolically unhealthy may also be beneficial [16].

Previous studies of the association between obesity, metabolic health, and depression were typically cross-sectional, yielding inconsistent results [4, 5, 8, 17]. As the association between obesity or metabolic disorders and depression was potentially bidirectional [3, 18], such study design may result in reverse causation. Nevertheless, our results of prospective analyses are in line with an individual-level meta-analysis of eight cross-sectional studies [5] and a nationwide study of inpatient data [17], suggesting that participants with metabolically healthy obesity were at higher risk of depression, and metabolically healthy obesity was associated with the highest risk. Another earlier study found that metabolically unhealthy obesity but not healthy obesity was associated with the risk of depression. However, this study has a much shorter follow-up period of 2 years than ours, with very few cases in subgroups. In addition, previous studies tend to use inconsistent definitions of metabolic health and allow for the presence of at least one metabolic risk factor for a healthy metabolic phenotype [4, 5, 8], which has been shown to be insufficient to define a benign obesity phenotype [10]. In line with previous study practice [7], we used a stricter definition of metabolic health, as the absence of three major metabolic disorders, to better define a healthy metabolic phenotype at potentially lower risk.

We found that diabetes and hypertension had a relatively stronger effect on depression for obese participants, aligning with a previous Mendelian randomization study indicating a potential causal relationship between depression and components of metabolic syndrome, specifically hypertension and fasting blood glucose [19]. However, the association of hypercholesterolemia with depression across BMI categories warrants further investigation, necessitating tailored study designs that account for additional confounding factors such as lipid-lowering drugs.

Although metabolic health may be a transient status and change over time [10, 11], most previous studies measured metabolic health in one single time point and failed to assess the health effect of changes in metabolic phenotypes [11]. About 50% of participants with metabolically healthy obesity converted to unhealthy phenotype over 8 years in the current study, which is in line with the percentage of 33–52% over 6–20 years in previous cohort studies [20–23]. We further demonstrated that such transition to unhealthy metabolic phenotype and longer exposure to metabolically unhealthy status are associated with a higher risk of depression; obese participants with initial metabolic health who progressed to unhealthy phenotype had a risk between those with stable metabolic health and those who were stable metabolically unhealthy. The association with metabolically healthy obesity was also stronger in a sub-sample of participants who were already obese about 5 years ago. When assessing the effect of baseline metabolic health, lower risks were observed for participants with metabolically healthy obese, possibly reflecting the misclassifications of healthy metabolic phenotype over time. The high conversion rate to unhealthy phenotypes may partly explain the inconsistent results in previous cross-sectional studies [8], which also suggest the importance of measuring metabolic conditions over time beyond single measures to better establish long-term depression risk.

The mechanism underlying the association between obesity, related metabolic abnormalities, and depression may involve the alterations in adipose-related metabolic signals, including glucocorticoids, adipose-derived hormones, and insulin and inflammatory cytokines [16, 24]. Endocrine changes related to central obesity include dysregulation of hypothalamic–pituitary–adrenal axis and alteration of the hormone levels (e.g., cortisol, leptin, and insulin), which are implicated in the control of mood and emotion. Obesity and related abnormalities may impair the brain glucocorticoids, leptin, and insulin receptor signaling that are linked to depressive symptoms. Inflammatory cytokines and signaling (e.g., interleukin-1β and C-reactive protein) stimulated by fat accumulation could promote depressive behavior through the neuroinflammatory pathway. Obesity is also associated with increased vulnerability to external stressors and negative emotional events. A recent Mendelian randomization study using metabolically favorable genetic variants associated with BMI suggested that metabolically healthy adiposity plays a causal role in developing depression [25], which supports our findings. Higher BMI may cause depression through multifaceted psychological and physiological pathways, partly independent of its adverse metabolic effects. Participants with persistent obesity and high metabolic risk are at the highest risk of depression, suggesting that obesity and metabolic abnormalities may have additive or synergistic effects on mental well-being. Integrated strategies for both weight control and prevention of obesity-related metabolic disorders should be informed to promote well-being and mental health. Strengths of the current study include the use of a nationally representative community-based cohort, the prospective design, long follow-up period, strict definition of metabolic health, and multiple sensitivity analyses to account for potential reverse causation and misclassification of both BMI and metabolic health. Additional strengths included an assessment of change in metabolic health status and its impact on subsequent of depression. However, several limitations are also worth noting. First, the current study population mainly consisted of White adults from the UK, which may limit the generalizability of our findings to other populations and settings. Second, although we used rigorous statistical methods and multiple sensitivity analyses to rule out the potential reverse causation caused by unhealthy normal weight, it’s likely that participants with undiagnosed metabolic diseases may be misclassified as being metabolically healthy. However, such misclassification tends to bias any genuine association with metabolically unhealthy obese towards the null. Third, we used a stricter definition of metabolic health different from the most common definitions based on individual components of metabolic syndrome in previous studies. This may make direct comparison difficult, however, the currently used definition was reported to better identify a benign obesity phenotype. Future studies are needed to compare the associations of metabolic health defined by various methods with the risk of depression. Fourth, although we adjusted for a set of covariates, and further excluded participants with diagnoses of mental disorders at baseline in the sensitivity analysis, residual confounding and reverse causality cannot be ruled out in this observational study. Fifth, as we included participants with available data on depression measured 12 years after the baseline at wave 8, the study population may be healthier. Finally, the study outcome of elevated depressive symptoms does not reflect clinically diagnosed depressive disorders, further studies based on clinical diagnosis of depression with repetitive measurements and long-term follow-up are needed to replicate our findings.

## Conclusion

Both obesity and common metabolic disorders are independently associated with an increased risk of depression. Obese participants without metabolic risk factors at baseline or maintained metabolically healthy over long periods of time remain at increased risk of depression, with the highest risk observed for those with metabolically unhealthy obesity. In addition, most participants with metabolic health converted to unhealthy metabolic phenotypes over time across BMI categories, which was associated with an increased risk of depression. Targeted behavioral interventions for the prevention of such adverse conversion in both obese and nonobese individuals, such as adherence to healthy diet and physical activity, may be beneficial to mental well-being.

## Supporting information

Zhu et al. supplementary material Zhu et al. supplementary material

## Data Availability

ELSA data were available through the UK Data Service (https://ukdataservice.ac.uk/).

## Supplementary material

The supplementary material for this article can be found at http://doi.org/10.1192/j.eurpsy.2024.20.
