# Supplementary material for: Transition in metabolic health phenotypes across general adiposity categories and association with the risk of depression: a prospective analysis
Source: Eur Psychiatry. 2024 Feb 29;67(1):e26. doi: 10.1192/j.eurpsy.2024.20 (PMC10988159; doi:10.1192/j.eurpsy.2024.20)
Supplement: Zhu et al. supplementary material [file S0924933824000208sup001.docx]

**Supplementary Content**

**Figure S1. Study design and timelines**


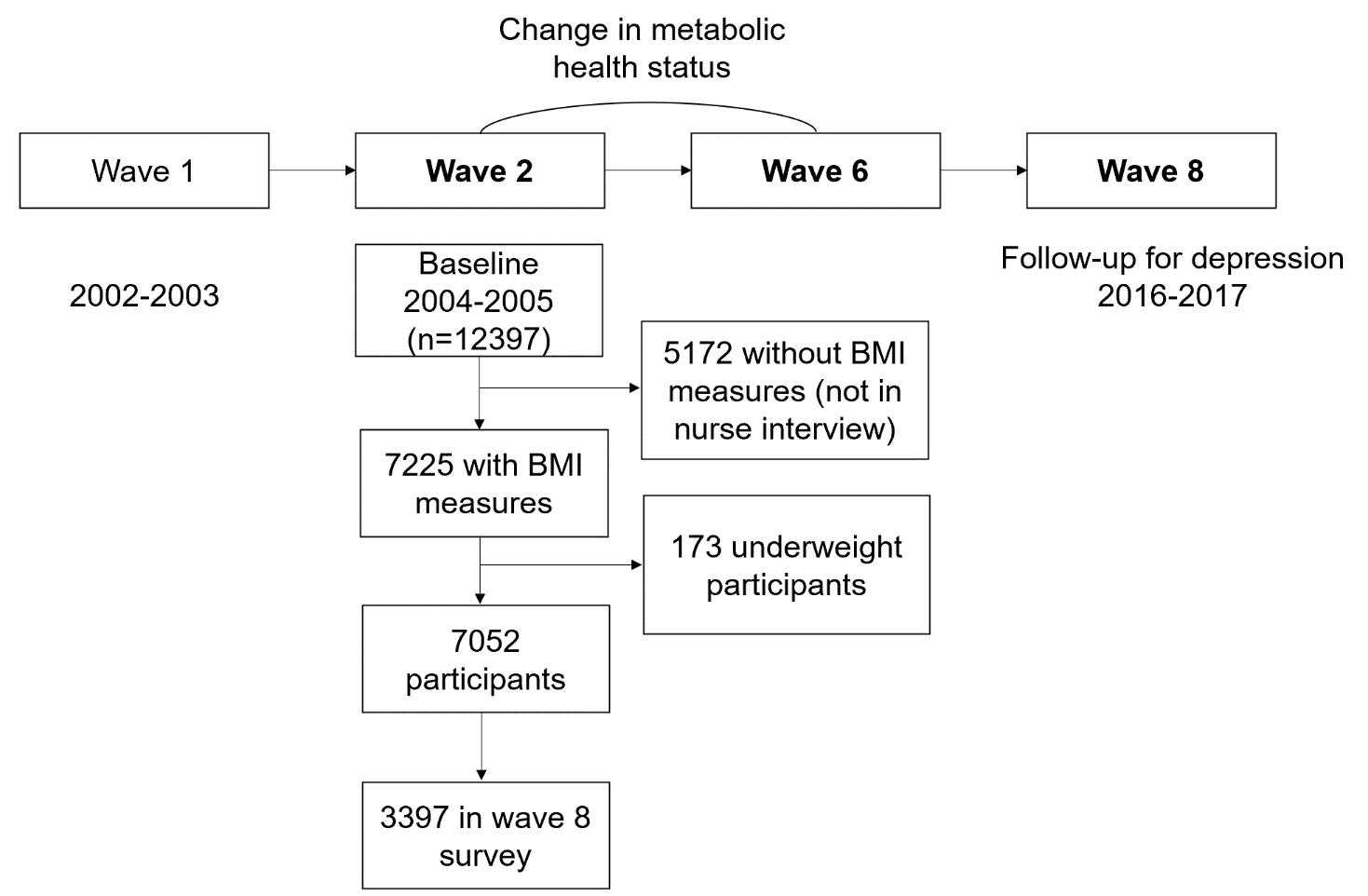


**Table S1. Subgroup analyses on the association general obesity, metabolic health, and risk of depression**

|  | **Non-obese** | | **Obese** | | ***P* value for interaction** |
| --- | --- | --- | --- | --- | --- |
|  | Metabolically healthy | Metabolically unhealthy | Metabolically healthy | Metabolically unhealthy |  |
| **Age** |  |  |  |  | 0.39 |
| <65 (n=2123) |  |  |  |  |  |
| OR, multivariate-adjusted^†^ | 1 [Reference] | 1.24 (0.82-1.86) | 1.73 (1.11-2.68) | 1.73 (1.13-2.63) |  |
| ≥65 (n=1274) |  |  |  |  |  |
| OR, multivariate-adjusted^†^ | 1 [Reference] | 1.24 (0.83-1.87) | 0.94 (0.49-1.71) | 1.51 (0.95-2.40) |  |
| **Sex** |  | |  | | 0.49 |
| Male (n=1463) |  |  |  |  |  |
| OR, multivariate-adjusted^†^ | 1 [Reference] | 1.77 (1.06-2.97) | 1.78 (0.87-3.48) | 2.27 (1.26-4.07) |  |
| Female (n=1934) |  |  |  |  |  |
| OR, multivariate-adjusted^†^ | 1 [Reference] | 1.11 (0.78-1.57) | 1.29 (0.85-1.95) | 1.44 (1.00-2.07) |  |
| **Smoking** |  | |  | | 0.99 |
| Never (n=1384) |  |  |  |  |  |
| OR, multivariate-adjusted^†^ | 1 [Reference] | 1.18 (0.74-1.85) | 1.56 (0.87-2.70) | 1.63 (0.98-2.70) |  |
| Ever (n=2013) |  |  |  |  |  |
| OR, multivariate-adjusted^†^ | 1 [Reference] | 1.31 (0.90-1.90) | 1.40 (0.87-2.20) | 1.61 (1.08-2.39) |  |

^†^Adjusted for age, sex, race/ethnicity, education level, smoking, alcohol consumption, physical activity, cardiovascular diseases, history of psychiatric disorders, CES-D score, and mutually for other metabolic risk factors at baseline.

**Table S2. Sensitivity analyses on the association general obesity, metabolic health, and risk of depression**

a, metabolic health at baseline

|  | **Non-obese** | | **Obese** | |
| --- | --- | --- | --- | --- |
|  | Metabolically healthy | Metabolically unhealthy | Metabolically healthy | Metabolically unhealthy |
| SA1: Excluding participants with diagnosed mental disorders at baseline (n=3186) | 1 [Reference] | 1.30 (0.96-1.75) | 1.38 (0.93-2.01) | 1.64 (1.18-2.28) |
| SA2: Further adjustment for other chronic diseases at baseline^†^ | 1 [Reference] | 1.24 (0.91-1.68) | 1.39 (0.95-2.01) | 1.64 (1.17-2.30) |
| SA3: Further adjustment for central obesity^#^ | 1 [Reference] | 1.24 (0.93-1.65) | 1.26 (0.84-1.88) | 1.43 (1.00-2.05) |
| SA4: Further adjustment for weight change between baseline at wave 2 and wave 6 | 1 [Reference] | 1.17 (0.86-1.60) | 1.34 (0.90-1.97) | 1.58 (1.12-2.21) |
| SA5: Using wave 9 data for the follow-up of depression (n=2950) | 1 [Reference] | 1.14 (0.84-1.53) | 1.14 (0.77-1.67) | 1.46 (1.04-2.03) |
| SA6: Excluding participants who were metabolically healthy obese at baseline but were not already obese at wave 0^*^ (n=3288) | 1 [Reference] | 1.26 (0.95-1.68) | 1.57 (1.06-2.30) | 1.62 (1.18-2.20) |

b, change in metabolic health between baseline and follow-up

|  | **Non-obese at baseline** | | | **Obese at baseline** | | |
| --- | --- | --- | --- | --- | --- | --- |
|  | Metabolically healthy at baseline | | Metabolically unhealthy at baseline | Metabolically healthy at baseline | | Metabolically unhealthy at baseline |
|  | Metabolically healthy at follow-up | Metabolically unhealthy at follow-up |  | Metabolically healthy at follow-up | Metabolically unhealthy at follow-up |  |
| SA1: Excluding participants with diagnosed mental disorders at baseline (n=3186) | 1 [Reference] | 1.38 (0.91-2.09) | 1.48 (1.04-2.12) | 1.64 (0.95 -2.76) | 1.52 (0.88-2.56) | 1.88 (1.29-2.76) |
| SA2: Further adjustment for other chronic diseases at baseline^†^ | 1 [Reference] | 1.48 (1.00-2.18) | 1.48 (1.06-2.09) | 1.67 (0.99-2.74) | 1.69 (1.03-2.74) | 1.88 (1.32-2.70) |
| SA3: Further adjustment for central obesity^#^ | 1 [Reference] | 1.46 (0.99-2.16) | 1.46 (1.04-2.05) | 1.48 (0.86-2.52) | 1.49 (0.88-2.49) | 1.69 (1.13-2.53) |
| SA4: Further adjustment for weight change between baseline at wave 2 and wave 6 | 1 [Reference] | 1.37 (0.90-2.08) | 1.34 (0.93-1.94) | 1.46 (0.80-2.56) | 1.58 (0.92-2.66) | 1.81 (1.23-2.67) |
| SA5: Using wave 9 data for the follow-up of depression (n=2950) | 1 [Reference] | 1.17 (0.78-1.74) | 1.21 (0.86-1.71) | 0.96 (0.52-1.70) | 1.45 (0.87-2.38) | 1.55 (1.07-2.25) |
| SA6: Excluding participants who were metabolically healthy obese at baseline but were not already obese at wave 0^*^ (n=3288) | 1 [Reference] | 1.48 (1.00-2.18) | 1.49 (1.07-2.09) | 1.70 (0.95-2.94) | 2.00 (1.17-3.33) | 1.91 (1.34-2.73) |

^†^Other chronic diseases included cardiovascular diseases, lung disease, asthma, arthritis, osteoporosis, cancer, Parkinson's disease, and dementia. Models were adjusted for age, sex, race/ethnicity, education level, smoking, alcohol consumption, physical activity, cardiovascular diseases, history of psychiatric disorders, CES-D score, and additionally for other covariates in sensitivity analyses.

^#^Central obesity as defined by waist circumference.

*73.5% of the metabolically healthy obese participants at baseline were in fact classified as obese already at wave 0.
